# Myricetin Induces Apoptosis and Protective Autophagy through Endoplasmic Reticulum Stress in Hepatocellular Carcinoma

**DOI:** 10.1155/2022/3115312

**Published:** 2022-05-29

**Authors:** Anlai Ji, Lili Hu, Ding Ma, Guanghui Qiang, Dongliang Yan, Guang Zhang, Chunping Jiang

**Affiliations:** ^1^Department of Hepatobiliary Surgery, Drum Tower Clinical College of Nanjing Medical University, Nanjing 210008, China; ^2^Department of General Surgery, The Affiliated Hospital of Yangzhou University, Yangzhou 225000, China; ^3^Jinan Microecological Biomedicine Shandong Laboratory, Shounuo City Light West Block, Qingdao Road 3716#, Huaiyin District, Jinan City, Shandong Province, China

## Abstract

Myricetin, a natural flavonoid, exhibits diverse biological activities, including antitumor effects. The present study aimed to investigate the effects of myricetin on hepatocellular carcinoma (HCC) cells and explore the underlying molecular mechanisms. Our results showed that myricetin significantly inhibited cell proliferation and induced apoptosis in HCC cells. The apoptosis induced by myricetin was associated with the activation of endoplasmic reticulum (ER) stress. In addition, autophagy was enhanced in response to ER stress. Inhibition of autophagy by RNA interference or chemical inhibitors resulted in increased apoptosis in myricetin-treated HCC cells. The in vivo experiment also showed that myricetin effectively reduced tumor growth in an HCC xenograft model and that combination treatment with an autophagy inhibitor significantly enhanced this effect. These results indicated that myricetin induced apoptosis in HCC cells through the activation of ER stress. Protective autophagy was also upregulated during this process. Simultaneous inhibition of autophagy enhanced the anti-HCC activity of myricetin. Myricetin might be a promising drug candidate for HCC therapy, and the combined use of myricetin with autophagy inhibitors could be an effective therapeutic strategy.

## 1. Introduction

Natural compounds are important resources for developing novel agents. Flavonoids, a group of natural polyphenolic compounds based on a 15-carbon skeleton and found ubiquitously in plants, are being increasingly studied for their various biological activities and negligible side effects [1]. Myricetin, one of the most common flavonoids that abounds in fruits, vegetables, herbs, tea, and red wine, has been found to have diverse biological activities, such as iron-chelating, antioxidant, anti-inflammatory, and antidiabetic effects, since it was discovered in 1896 [2]. Moreover, myricetin shows anticancer effects [3–5].

Hepatocellular carcinoma (HCC) is the most frequently diagnosed primary liver cancer, accounting for more than 90% of all cases [6]. In 2012, HCC caused more than 780000 cases and 740000 deaths worldwide. Surgical resection, transplantation, and transarterial chemoembolization have proven survival benefits for patients with early-stage disease [6]. However, most patients are diagnosed at a late stage and are not candidates for radical therapies. For patients with advanced-stage disease, systemic chemotherapy exhibits marginal activity with associated toxicity and provides no survival benefit [7]. The tyrosine kinase inhibitors sorafenib, regorafenib, and lenvatinib can improve overall survival as first- or second-line therapies [8–10]. However, the development of drug resistance remains the major obstacle to better outcomes in HCC patients. Thus, the discovery of novel therapeutic agents is in urgent demand.

Previous studies have shown that myricetin can selectively induce apoptosis of cancerous hepatocytes induced by diethylnitrosamine (DEN) without cytotoxicity in normal hepatocytes in rats [11]. In human HCC cell lines, myricetin can induce cell cycle arrest by inactivating cell cycle-related proteins and inhibiting cyclin-dependent kinase 1 (CDK1) activity or induce apoptosis through the mitochondrial pathway by reducing the mitochondrial membrane potential and downregulating antiapoptotic proteins and upregulating proapoptotic proteins [5, 12]. These preliminary results suggest that myricetin may be a potential agent for the treatment of HCC, and further research, especially in vivo studies, is urgently needed. Therefore, the current study was designed to use both in vitro and in vivo experiments to investigate the effects of myricetin on HCC cells and explore the underlying molecular mechanisms.

## 2. Materials and Methods

### 2.1. Compounds

Myricetin (Selleck Chemicals, Shanghai, China), BAPTA-AM (Selleck Chemicals, Shanghai, China), and tunicamycin (Sigma Aldrich, St. Louis, USA) were dissolved in 100% dimethyl sulfoxide (DMSO). Chloroquine (CQ, Sigma Aldrich, St. Louis, USA) was dissolved in PBS. Chemicals were further diluted with cell culture medium for in vitro studies or PBS for in vivo studies to the indicated concentration.

### 2.2. Cell Culture

The human hepatocyte cell line HL-7702 and human HCC cell lines SMMC-7721 and Hep3B were purchased from the Cell Bank of Xiangya Central Experiment Laboratory of Central South University (Changsha, China). HL-7702 cells were cultured in RPMI-1640 (WISENT, CA, USA) supplemented with 10% fetal bovine serum (FBS, WISENT, CA, USA), whereas SMMC-7721 and Hep3B cells were cultured in Dulbecco's modified Eagle's Medium (DMEM, WISENT, CA, USA) containing 10% fetal bovine serum (FBS, WISENT, CA, USA). All cells were cultured in a humidified atmosphere at 37°C with 5% CO_2_.

### 2.3. Cell Viability Assay

Cell viability was determined using a Cell Counting Kit-8 (CCK-8) assay according to the manufacturer's instructions. Generally, 5 × 10^3^ cells were added to 96-well plates. Subsequently, the cells were treated with different doses of myricetin for 12, 24, and 48 h. Then, the medium of each well was removed, and 100 *µ*l of fresh culture medium with 10% CCK-8 was added to each well for further incubation for an additional 3 h. The absorbance was measured at 450 nm with a microplate reader. All experiments were repeated three times.

### 2.4. RNA Interference

Oligonucleotides were synthesized at GenePharma (Shanghai, China) and transfected into cells using Lipofectamine 2000 (Invitrogen, USA). Sequences for the C/EBP homologous protein (CHOP), IRE1*α,* and ATG5 siRNAs were described in a previous study by Wang et al. [13].

### 2.5. Flow Cytometry

The cells (5 × 10^6^/well) were seeded into 6-well plates and then treated with different doses of myricetin for 24 h. The cells, including adherent, apoptotic, and dead cells, were harvested. After washing with cold PBS, Annexin V-FITC binding buffer was added to resuspend the cells. After staining with Annexin V-FITC/propidium iodide (PI), apoptosis was measured by flow cytometry (FACSCalibur, BD, USA).

### 2.6. Western Blot Analysis

To determine the level of the indicated proteins, whole-cell protein was collected from the cells using radioimmunoprecipitation assay (RIPA) lysis buffer (Beyotime Biotechnology, China) with 1% protease inhibitors (Thermo Scientific, USA). The concentration of protein was determined by a BCA protein assay kit (Thermo Scientific, USA). Then, the proteins were separated by sodium dodecyl sulfate–polyacrylamide gel electrophoresis (SDS–PAGE). Antibodies against GAPDH, caspase-3, cleaved caspase-3, caspase-9, cleaved caspase-9, PARP, cleaved PARP, CHOP, p62, JNK, p-JNK, IRE1*α,* and ATG5 were purchased from Cell Signaling Technology (Beverly, MA, USA). An antibody against LC3 was purchased from Sigma Aldrich (St. Louis, USA). The protein bands were measured through chemiluminescence (ECL) reagent (Millipore, Bedford, MA, USA) after incubation with HRP-conjugated secondary antibodies at room temperature for 2 h. Then, they were imaged directly using a FluorChem FC2 Imaging System (Alpha Innotech, San Leandro, CA, USA), and the relative density of LC3-II/LC3-I bands was calculated using ImageJ 1.48v software.

### 2.7. Confocal Microscopy

SMMC-7721 and Hep3B cells (1 × 10^5^ per well) were grown on sterile coverslips in 12-well plates. Then, the cells were transfected with GFP-LC3 using Lipofectamine 2000 (Invitrogen, USA) at 37°C for 4 h in accordance with the manufacturer's instructions. After 12 h, the cells were treated with different doses of myricetin for 24 h. Subsequently, the medium of each well was removed, and the cells were washed with cold PBS three times and further fixed with 4% paraformaldehyde at room temperature for 15 min. Then, the cells were washed with PBS three times again and examined with a confocal microscope (FV10i, Olympus, Japan).

### 2.8. Animal Model

All animal studies were performed according to the national guidelines of the Institutional Animal Care and Use Committee (IACUC) and were approved by the Ethics Committee of Nanjing Drum Tower Hospital. Four- to six-week-old male nude mice were obtained from the Model Animal Research Center of Nanjing University. First, five mice received subcutaneous injections of SMMC-7721 cells into the left axillary region of mice (1 × 10^7^ cells in 200 *μ*L PBS per mouse). When the tumors grew to approximately 200 mm^3^, the animals were sacrificed, and the tumors were collected. Then, the tumors were cut into cubes (2 × 2 × 2 mm in size) and were merged in cold serum-free medium. For the orthotopic hepatocellular carcinoma model, the tumors were cut into cubes and transplanted into the left liver lobe of 20 male nude mice under infiltration anesthesia with isoflurane. Finally, the liver was sutured and returned to the abdominal cavity. Fourteen days after tumor transplantation, the mice were divided randomly into four groups (*n* = 5; the control group, myricetin group, CQ group, and myricetin + CQ group). The myricetin group and myricetin + CQ group received 30 mg/kg myricetin daily by intraperitoneal injection. The CQ group and myricetin + CQ group received 60 mg/kg CQ daily by intraperitoneal injection. After 15 days of intraperitoneal injection, the liver tissues were collected from mice, and the tumor sizes were measured using calipers. Tumor volume was calculated as V = width^2^ × length/2. Then, tumors were fixed in 4% PFA for TUNEL staining.

### 2.9. In Vivo TUNEL Apoptosis Assay

The tumors from nude mice were paraffin-sectioned by routine methods. The fixed cells were incubated in 1.0 *µ*g/mL proteinase K for 20 min at room temperature and then washed with PBS three times, followed by incubation with the reaction solution at 37°C for 60 min. The tissue sections were washed with PBS three times after removal of the reaction solution and were incubated with HRP-conjugated antidigoxin antibody for 30 min at room temperature. Finally, the cells were incubated with DAB solution. The images were acquired by using an Olympus microscope with a digital camera.

### 2.10. Statistical Analysis

All data are expressed as the mean ± SD. Student's *t*-test or one-way ANOVA with Dunnett's test was performed to assess the differences between groups. *P* values less than 0.05 were considered statistically significant.

## 3. Results

### 3.1. Myricetin Inhibited Proliferation and Induced Apoptosis in Human Hepatocellular Carcinoma Cells

**Figure 1:**
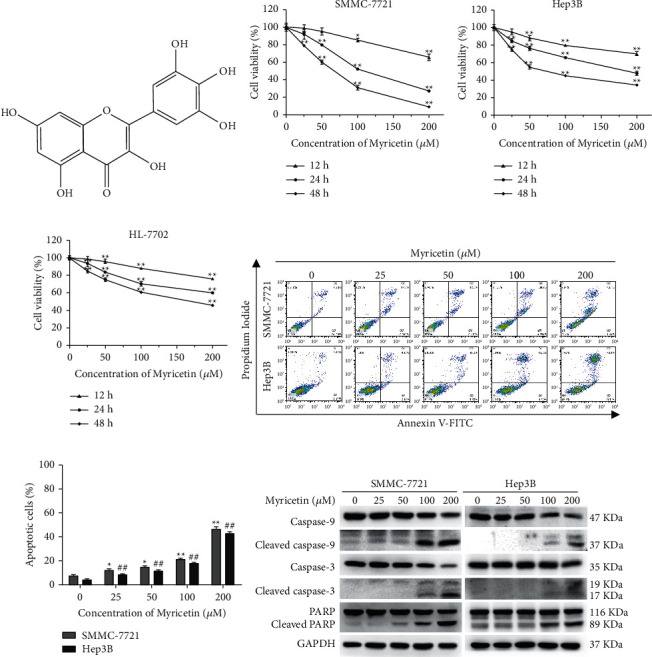
Myricetin induces apoptosis in human hepatocellular carcinoma cells. (a) The chemical structure of myricetin. (b, c, d) The human HCC cell lines SMMC-7721 (b) and Hep3B (c) and the human normal hepatocyte cell line HL-7702 (d) were treated with 0, 25, 50, 100, or 200 *μ*M myricetin for 12 h, 24 h, and 48 h. The viability of the cells was examined with a CCK-8 assay. Data are expressed as the percentage over the untreated control. Values are expressed as the mean ± S.D. (e, f). SMMC-7721 and Hep3B cells treated with various concentrations of myricetin for 24 h were subjected to flow cytometry to measure cell apoptosis after staining with Annexin V/PI. Data from cell apoptosis shown are representative of three independent experiments, and histograms are shown for analyzed cells. (g) SMMC-7721 and Hep3B cells were treated with various concentrations of myricetin for 24 h and the expression of cleaved PARP, caspase-3, cleaved caspase-3, caspase-9, cleaved caspase-9, PARP, and cleaved PARP was detected by western blotting. Data shown are the means ± S.D. from 3 independent experiments. ^*∗*^^/#^*P* < 0.05, ^*∗∗*^^/##^*P* < 0.01.

To investigate the cytotoxic effect of myricetin (Figure 1(a)) on human hepatocellular carcinoma cells, two HCC cell lines, SMMC-7721 and Hep3B, were treated with myricetin at a series of different concentrations (0, 25, 50, 100, and 200 *μ*M) for 12 h, 24 h, and 48 h, and cell viability was assessed by CCK-8 assay. As shown in Figures 1(b) and 1(c), myricetin significantly suppressed the proliferation of HCC cells in a dose- and time-dependent manner. The IC50 values were 109.56 *μ*M (24 h) and 58.3 *μ*M (48 h) for SMMC-7721 cells, and the IC50 values were 196.4 *μ*M (24 h) and 81.1 *μ*M (48 h) for Hep3B cells. To validate whether the cytotoxic effect of myricetin on human hepatocellular carcinoma cells is cancer-selective, the cytotoxic effect was evaluated. The cell line HL-7702, a human normal hepatocyte cell line, was treated with myricetin, and the IC50 values were 252.2 *μ*M (24 h) and 163.9 *μ*M (48 h), which were significantly higher than those of SMMC-7721 and Hep3B cells. As shown in Figure 1(d), the inhibitory effect of myricetin on HL-7702 cells was apparently weaker than that on SMMC-7721 and Hep3B cells. Therefore, myricetin showed partial cancer-selective inhibition in human HCC cells, while the cytotoxicity in a normal human hepatocyte cell line was relatively lower.

Next, to verify the mechanism of myricetin in suppressing HCC cells, SMMC-7721 and Hep3B cells were exposed to myricetin for 24 h and then subjected to flow cytometry to measure cell apoptosis after being stained with Annexin V/PI. As illustrated in Figures 1(e) and 1(f), the percentage of cells undergoing apoptosis (the upper-right quadrant) increased in a dose-dependent manner. The activity of classical apoptotic proteins in cells was also examined by incubation with myricetin for 24 h by western blotting. As shown in Figure 1(g), a significant increase in the activation of cleaved caspase-9 and -3 as well as PARP was observed. These results demonstrated that myricetin displayed a specifically profound antiproliferative effect on HCC cells, and such proliferation inhibition is correlated with apoptosis induced by myricetin.

### 3.2. Endoplasmic Reticulum (ER) Stress Was Induced by Myricetin and Participated in Myricetin-Induced Apoptosis in Human Hepatocellular Carcinoma Cells

**Figure 2:**
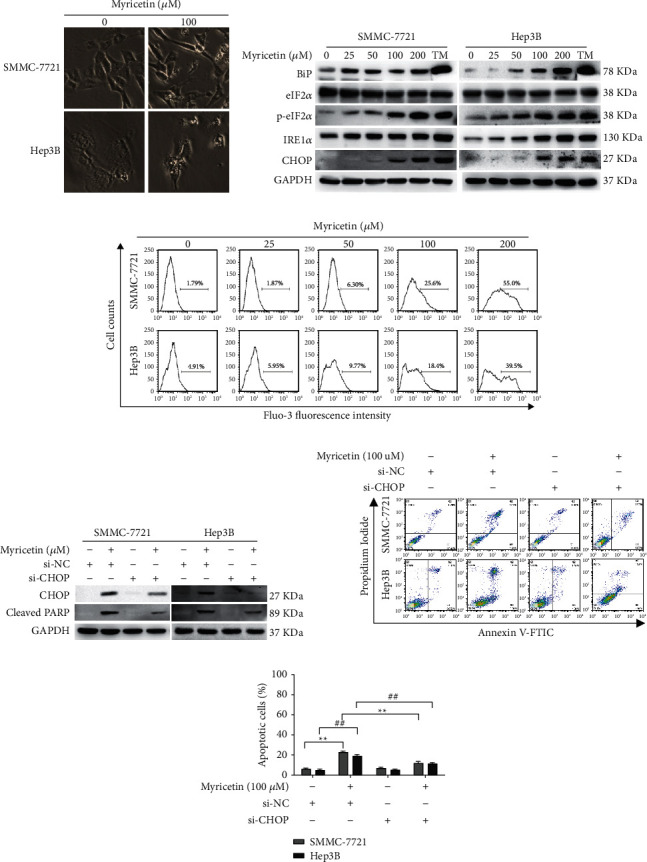
Myricetin-mediated ER stress participated in myricetin-induced apoptosis in human hepatocellular carcinoma cells. (a) Morphological changes in SMMC-7721 and Hep3B cells were observed under an inverted light microscope (100×) after treatment with 100 *μ*M myricetin for 24 h. (b). SMMC-7721 and Hep3B cells were incubated with various concentrations of myricetin and tunicamycin (5 *µ*g/ml) for 24 h and the expression of BiP, eIF2*α*, p-eIF2*α*, IRE1*α,* and CHOP was detected by western blotting. (c) SMMC-7721 and Hep3B cells were treated with various concentrations of myricetin for 24 h. Then, the level of intracellular Ca^2+^ was measured using flow cytometry with Fluo-3 AM staining. (d) Control siRNA or CHOP siRNAs were transfected into SMMC-7721 and Hep3B cells. After cells were exposed to vehicle or 100 *μ*M myricetin for 24 h, the protein levels of PARP and CHOP were validated by western blotting. (e) (f). Cells were treated and then harvested for apoptosis detection using Annexin V and PI staining with flow cytometry. Representative data from three independent experiments in triplicate are shown as the mean ± S.D. ^*∗*^^/#^*P* < 0.05, ^*∗∗*^^/##^*P* < 0.01.

Cellular vacuolization, which may be dilated ER lumens, was also observed in HCC cells after treatment with myricetin (Figure 2(a)). To further evaluate whether myricetin induces ER stress in HCC cells, the expression levels of several molecular indicators of ER stress, including BiP, eIF2*α*, p-eIF2*α*, IRE1*α,* and CHOP, were checked by western blotting after SMMC-7721 and Hep3B cells were treated with various concentrations of myricetin for 24 h. Tunicamycin, a well-characterized ER stress inducer, was used as a positive control. The levels of BiP, a chaperone protein that binds to the luminal domain of ER transmembrane proteins to keep them inactive; IRE1*α*, an ER transmembrane protein that, when activated, induces signal transduction to upregulate the transcription of genes involved in protein folding, ER biogenesis, and ER-associated degradation; p-eIF2*α*, the activated form of eIF2*α*, which is phosphorylated by the ER transmembrane protein PERK to inhibit polypeptide chain synthesis and the ER stress downstream protein CHOP increased in a dose-dependent manner (Figure 2(b)).

Another important function of the ER is as an intracellular reservoir for Ca^2+^. Ca^2+^ may be released from the ER to the cytosol when ER homeostasis is disrupted. Ca^2+^ mobilization was analyzed. As shown in Figure 2(c), myricetin induced a prominent elevation in cytoplasmic calcium levels. Taken together, these results indicated that myricetin treatment could induce ER stress in HCC cells.

Sustained ER stress can cause cell death. To determine the role of ER stress in myricetin-induced apoptosis, CHOP, which can be induced by all three ER stress sensors and plays an important role in ER stress-mediated apoptosis, was silenced by a specific siRNA (si-CHOP). Western blotting showed that the siRNA efficiently prevented the increase in CHOP expression after myricetin treatment in HCC cells and that the silencing of CHOP reduced the level of cleaved PARP (Figure 2(d)). Apoptosis was further examined by Annexin V/PI staining. We found that the percentage of apoptotic cells decreased in cells treated with myricetin and si-CHOP together compared with cells exposed to myricetin treatment alone (Figures 2(e) and 2(f)). These data indicated that myricetin-induced apoptosis was at least partly due to ER stress.

### 3.3. Myricetin Induced ER Stress-Mediated Autophagy in Human Hepatocellular Carcinoma Cells

**Figure 3:**
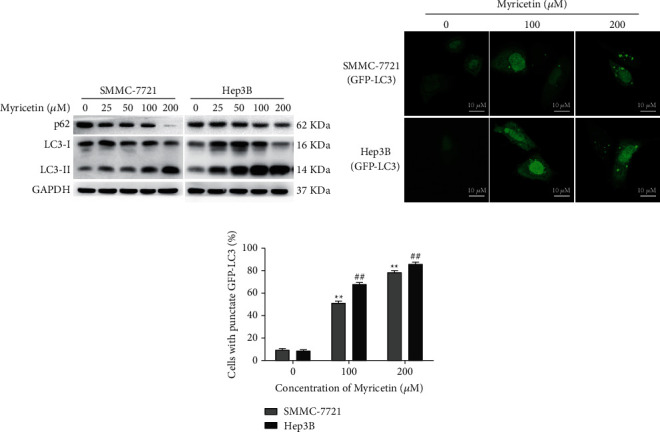
Myricetin induced autophagy in human hepatocellular carcinoma cells. (a) SMMC-7721 and Hep3B cells were treated with various concentrations of myricetin for 24 h, and the expression of p62, LC3-I, and LC3-II was detected by western blotting. (b, c). SMMC-7721 and Hep3B cells transfected with GFP-LC3 plasmids were treated with 0, 100, or 200 *μ*M myricetin for 24 h. Then, the cells were fixed with 4% PFA for confocal microscopy. Representative fluorescence images are shown in (b). The bar in the diagrams indicates 10 *µ*M length. The percentage of cells presenting typical GFP-LC3 puncta was quantified. (c) At least 100 cells were quantified from each experiment, and the data are presented as the mean ± S.D. from three independent experiments. ^*∗*^^/#^*P* < 0.05, ^*∗∗*^^/##^*P* < 0.01.

Whether myricetin induced autophagy in HCC cells was investigated next. The hallmark protein of autophagy, LC3, which has two forms, LC3-I and LC3-II, and p62 in SMMC-7721 and Hep3B cells were checked by western blotting after 24 h of treatment with myricetin. During the initiating step of autophagy, LC3-I binds to phosphatidylethanolamine and converts to the form LC3-II. Consequently, the conversion of LC3-I to LC3-II (ratio of LC3-II/LC3-I) is usually used as a marker of autophagy. The p62 protein is a selective substrate of autophagy, and decreased p62 levels are always associated with autophagy activation. As shown in Figure 3(a), the ratio of LC3-II/LC3-I was significantly increased, and the protein level of p62 was obviously reduced in a dose-dependent manner. To further confirm this result, SMMC-7721 and Hep3B cells transfected with GFP-LC3 plasmids and stably expressing GFP-LC3 were treated with 0 *μ*M, 100 *μ*M, and 200 *μ*M myricetin for 24 h, and GFP-LC3 punctate accumulation in cells was then monitored by confocal microscopy. As shown in Figures 3(b) and 3(c), the percentage of cells forming GFP-LC3 puncta was significantly increased in a dose-dependent manner. These data indicated that myricetin induced autophagic flux in HCC cells.

**Figure 4:**
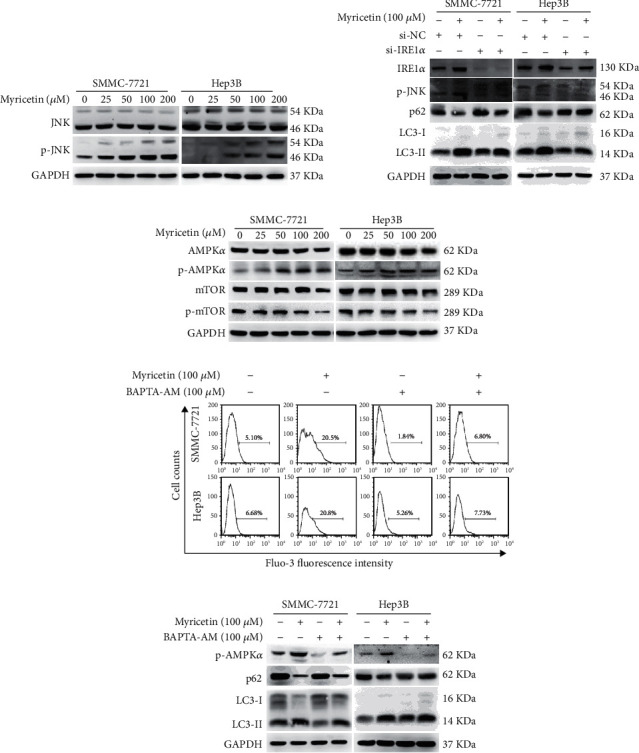
Myricetin-induced autophagy is mediated by ER stress activation. (a) SMMC-7721 and Hep3B cells were treated with various concentrations of myricetin for 24 h, and the expression of JNK and p-JNK was detected by western blotting. (b) SMMC-7721 and Hep3B cells were transfected with negative control siRNA or specific IRE1*α* siRNA and treated with 100 *μ*M myricetin for 24 h. IRE1*α*, p-JNK, p62, LC3-I, and LC3-II expression was observed by western blotting. (c) SMMC-7721 and Hep3B cells were treated with various concentrations of myricetin for 24 h. The expression of AMPK*α*, p-AMPK*α*, mTOR, and p-mTOR was detected by western blotting. (d) SMMC-7721 and Hep3B cells were pretreated with 10 *μ*M BAPTA-AM for 2 h and then exposed to 100 *μ*M myricetin or vehicle for 24 h. Then, the cells were loaded with Fluo-3 AM for flow cytometry analysis. (e) The cells were treated as in (d) and checked for the expression of p-AMPK, p62, LC3-I, and LC3-II by western blotting.

The protein expression of IRE1*α* was significantly increased after HCC cells were treated with myricetin. Meanwhile, the phosphorylation of JNK was also increased in a dose-dependent manner (Figure 4(a)), which indicated that JNK was activated after myricetin treatment. To clarify whether JNK was activated by IRE1*α* and subsequently activated autophagy in HCC cells, SMMC-7721 and Hep3B cells were transfected with IRE1*α* siRNA (si-IRE1*α*) or negative control siRNA (si-NC) and then incubated with 100 *μ*M myricetin or vehicle for 24 h. As shown in Figure 4(b), the expression of IRE1*α* was significantly decreased by siRNA, and in cells transfected with si-IRE1*α*, the phosphorylation of JNK, the conversion of LC3-I to LC3-II and the degradation of p62 were decreased compared with those in cells transfected with si-NC after treatment with myricetin, indicating that IRE1*α* knockdown inhibited the activation of JNK and autophagy.

The Ca^2+^-AMPK pathway is another common signal transduction pathway involved in ER stress-activated autophagy. We examined the downstream signaling molecules, and activation of AMPK*α* and inhibition of mTOR were observed (Figure 4(c)), indicating that the Ca^2+^-AMPK pathway may also take part in myricetin-induced autophagy. To confirm this hypothesis, we used BAPTA-AM, a calcium chelator, to overcome cytoplasmic Ca^2+^ elevation. SMMC-7721 and Hep3B cells were pretreated with 10 *μ*M BAPTA-AM for 2 h, followed by treatment with 100 *μ*M myricetin for another 24 h. As shown in Figure 4(d), cytoplasmic Ca^2+^ elevation was eliminated in BAPTA-AM-pretreated cells. Meanwhile, AMPK and autophagy activation were inhibited (Figure 4(e)). Taken together, these data demonstrated that ER stress activated complete autophagy through, at least in part, the IRE1*α*-JNK and Ca^2+^-AMPK pathways in HCC cells treated with myricetin.

### 3.4. Blocking Autophagy Enhanced the Apoptosis-Inducing Effect of Myricetin in Hepatocellular Carcinoma Cells

**Figure 5:**
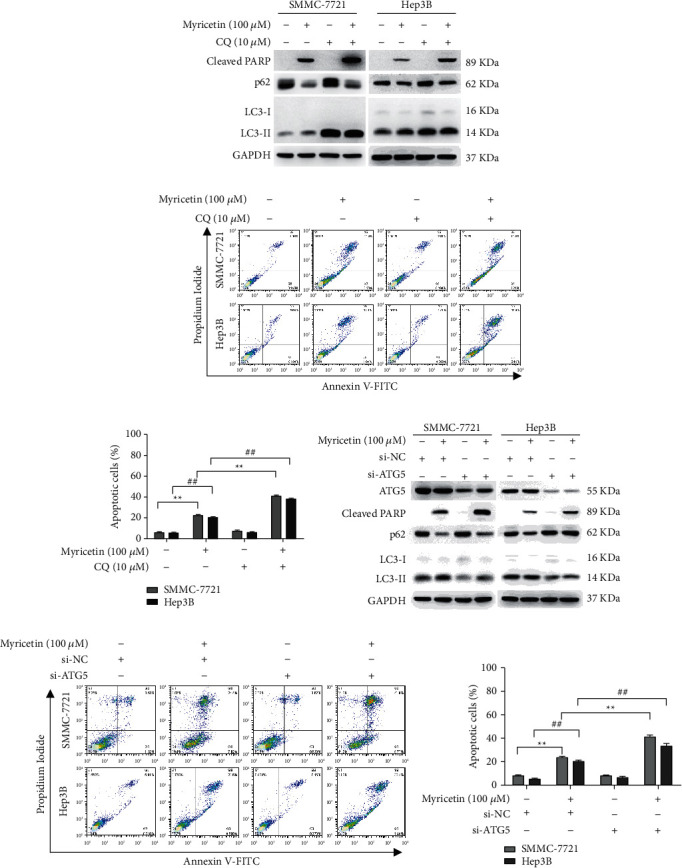
Blocking autophagy enhanced the apoptosis effect of myricetin on human hepatocellular carcinoma cells. (a) SMMC-7721 and Hep3B cells were treated with 100 *μ*M myricetin for 24 h in the presence or absence of 10 *μ*M CQ and observed for cleaved PARP, p62, LC3-I, and LC3-II expression by western blot analysis. (b, c). Cells treated as in (a) were assayed for apoptosis using Annexin V and PI staining with flow cytometry. Data were generated from three independent experiments and are shown as the mean ± S.D. (d) SMMC-7721 and Hep3B cells transfected with negative control siRNA or with ATG5 siRNA were treated with 100 *μ*M myricetin or vehicle for 24 h, and the expression of ATG5, p62, cleaved PARP, LC3-I, and LC3-II was detected by western blotting. (e, f). Cells treated as in (d) were assayed for apoptosis using Annexin V and PI staining with flow cytometry. Data were generated from three independent experiments and are shown as the mean ± S.D. ^*∗*^^/#^*P* < 0.05, ^*∗∗*^^/##^*P* < 0.01.

We used CQ, an autophagy inhibitor that can prevent autophagic degradation by interfering with lysosomal acidification and therefore inhibiting the activity of degradative enzymes, and a specific siRNA against ATG5, which is essential for the elongation of the autophagosome membrane to block the autophagy process. SMMC-7721 and Hep3B cells were treated with 100 *μ*M myricetin either alone or in the presence of 10 *μ*M CQ for 24 h. As shown in Figure 5(a), the level of p62 was restored, and the ratio of LC3-II/LC3-I was increased due to the accumulation of ineffective autophagosomes in myricetin- and CQ-cotreated cells. Cell apoptosis was monitored by the level of the apoptosis-related protein cleaved PARP and Annexin V/PI staining. The levels of cleaved PARP and apoptotic cells were markedly increased in cotreated cells compared with those in cells treated with myricetin only (Figures 5(a)–5(c)).

SMMC-7721 and Hep3B cells were transfected with ATG5 siRNA (si-ATG5) or negative control siRNA and then exposed to 100 *μ*M myricetin or vehicle for 24 h. Autophagy induced by myricetin was significantly inactivated by si-ATG5, as evidenced by the inhibition of p62 degradation and LC3 conversion (Figure 5(d)). Inhibition of autophagy in HCC cells exposed to myricetin by si-ATG5 also significantly enhanced myricetin-induced apoptosis (Figures 5(d)–5(f)). Taken together, these data showed that inhibition of autophagy induced by ER stress enhanced the antitumor effect of myricetin.

### 3.5. Myricetin Suppressed the Growth of HCC In Vivo, and the Effect Was Enhanced by CQ

**Figure 6:**
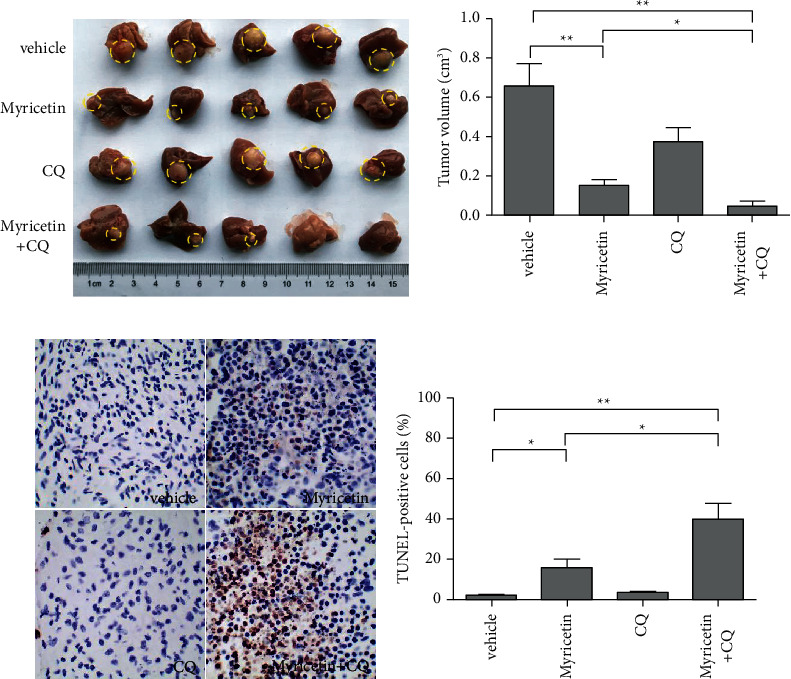
Effects of myricetin and CQ on orthotopic SMMC-7721 HCC tumors. (a, b). Livers from mice in each group 5 weeks after tumor implantation are shown. The average tumor volume for each group was calculated. Data are expressed as the mean ± S.D. (c, d). Apoptosis was measured by TUNEL staining of tumor tissue sections. TUNEL-positive cells were quantified using microscopy at magnification (400×) in eight fields for each tumor sample. Data are expressed as the mean ± S.D. ^*∗*^*P* < 0.05, ^*∗∗*^^/^*P* < 0.01.

We further investigated the antitumor activities of myricetin on HCC and the effect of autophagy on this process in vivo. An orthotopic xenograft model of HCC in nude mice derived from SMMC-7721 cells was developed and divided into four groups: mice treated with vehicle only, mice treated with myricetin only, mice treated with CQ only, and mice cotreated with myricetin and CQ. After the treatment, the results showed that myricetin markedly decreased the tumor volume compared with that in the vehicle control group. Furthermore, combination with CQ further enhanced the myricetin-mediated decrease in tumor size (Figures 6(a) and 6(b)). Meanwhile, immunochemistry assays of harvested tumors showed increased TUNEL-positive cells in tumors treated with myricetin compared with vehicle, and significantly more increased TUNEL-positive cells were observed in cotreated tumors in comparison with the myricetin-treated tumors (Figures 6(c) and 6(d)). Collectively, these results indicated that myricetin also established a good ability to induce apoptosis in HCC in vivo and that autophagy inhibition enhanced cytotoxicity.

## 4. Discussion

Myricetin, a common and prevalent herbal flavonoid, plays beneficial roles in numerous human diseases, including cardiovascular pathologies, inflammation, and cancers [14, 15]. Especially in recent years, numerous reports have shown that myricetin can modulate cancer development and progression by influencing distinct cell functions, including cell proliferation [5], autophagy [16], apoptosis [17], angiogenesis [18], drug chemosensitivity [19], and DNA replication and repair [20]. For example, the ability of myricetin to inhibit cell proliferation and induce apoptosis has been observed in many kinds of cancers, such as gastrointestinal cancer [21], colon cancer [20], ovarian cancer [4], breast cancer [22], and liver cancer [17]. In our study, the CCK-8 assay indicated that myricetin significantly suppressed the viability of HCC cells in a dose- and time-dependent manner with relative cancer specificity since the viability inhibition in the normal human hepatocyte cell line was mild and limited (Figures 1(b)–1(d)). Further investigations revealed that consistent with a previous report [17], myricetin induced apoptosis in SMMC-7721 and Hep3B HCC cell lines, as indicated by the activation of the caspase-9, caspase-3, and PARP signaling cascades (Figure 1(g)). In previous studies, it has also been found that myricetin causes HepG2 cell cycle arrest by inactivating the cyclin B/CDK1 complex and inhibiting CDK1 activity in HCC cells [5]. Myricetin could also induce apoptosis in HCC cells by reducing the mitochondrial membrane potential, thus releasing cytochrome C from the mitochondria and triggering the activation of caspase-3 and poly-adenosine diphosphate (ADP)-ribose polymerase 1 (PARP-1) [12]. In addition, myricetin downregulated antiapoptotic proteins, such as Bcl-2 and Bax, and upregulated the proapoptotic protein Bad [3]. Overall, previous studies and our present results confirmed that myricetin inhibited cancer cells, probably through the induction of apoptosis and cell cycle arrest. However, the detailed mechanisms underlying the inhibition of HCC cells by myricetin remain incompletely understood.

Diverse cellular stresses can cause ER stress, which is characterized by the accumulation of unfolded or misfolded proteins in the ER lumen [23]. ER stress can be buffered by the UPR process to orchestrate the recovery of ER function and restore homeostasis. When ER stress persists or cannot be recovered, UPR may also lead to cell death through various signaling cascades [24]. Numerous reports have shown that small molecules can lead to disordered protein maturation and folding, which in turn leads to the accumulation of aberrant proteins and UPR in the ER. For example, resveratrol could induce ER stress-mediated apoptosis in ovarian cancer cells [25]. In this study, we report that myricetin induced ER stress in HCC cells, as indicated by typical cellular morphology (Figure 2(a)), the release of intracellular calcium and the activation of ER stress and UPR signaling molecules, including BiP, eIF2*α*, IRE1*α,* and CHOP (Figure 2(b)). Using an RNA interference technique, we silenced the expression of CHOP, which was reported to mediate ER stress-induced apoptosis [26]. CHOP interference significantly attenuated myricetin-induced apoptosis, indicating that this protein was the mediator of ER stress-related cell death (Figure 2(d)). Although the specific target of myricetin is still unclear, our study provided preliminary evidence that myricetin-induced HCC cell apoptosis was at least partially mediated by ER stress and downstream CHOP signaling.

Our results also found that myricetin induced protective autophagy in HCC cells. Autophagy is a major catabolic process that is responsible for endolysosomal degradation of misfolded proteins, detrimental cellular substances, defective organelles, and pathogens. Autophagy is well orchestrated by more than thirty autophagy-related genes (ATGs), which can be activated by nutrient starvation and inhibition of mTOR signaling [27]. Increasing evidence has shown the presence of crosstalk between ER stress and autophagy. Autophagy can be triggered by ER stress. ATF4 and CHOP transcriptionally regulate a small number of ATG genes, while the IRE1*α*-JNK and Ca^2+^-AMPK pathways also regulate autophagy [28, 29]. Our results showed that myricetin strongly activated autophagy in HCC cells, as indicated by the degradation of p62, the conversion of LC-3, and the formation of autophagosomes (Figures 3(a) and 3(b)). Recently, a report showed that the inhibition of mTOR phosphorylation by myricetin leads to autophagy [16]. We found that myricetin treatment induced autophagy by influencing ER stress and UPR signaling pathways, including IRE1*α*-JNK and Ca^2+^-AMPK, which may act as upstream regulators of mTOR [30, 31]. The IRE1*α*-JNK pathway was significantly activated after myricetin-induced ER stress. However, siRNA knockdown of IRE1*α* in HCC cells led to inhibited phosphorylation of JNK, less conversion from LC3-I to LC3-II, and decreased degradation of p62 compared with those in cells transfected with control scramble RNAs under myricetin treatment (Figure 4(b)). These results indicated that the IRE1*α*-JNK pathway represented an activator of autophagy during myricetin-induced ER stress. We further investigated whether the Ca^2+^-AMPK pathway also participates in myricetin-induced autophagy. Myricetin treatment markedly activated AMPK and subsequently inhibited the phosphorylation of mTOR (Figure 4(c)), which is consistent with the study by Cao et al. [16]. BAPTA-AM, a calcium chelator, was used to block cytoplasmic Ca^2+^ elevation. In BAPTA-AM-pretreated HCC cells, the elevation of cytoplasmic Ca^2+^ induced by myricetin treatment was attenuated. Meanwhile, the phosphorylation of AMPK and the activation of autophagy were also inhibited by cotreatment of BAPTA-AM with myricetin compared with myricetin treatment alone (Figures 4(d) and 4(e)), indicating that Ca^2+^-AMPK signaling may also participate in ER stress-induced autophagy during myricetin treatment. Taken together, these data demonstrated that ER stress activated autophagy, at least partially, through the IRE1*α*-JNK and Ca^2+^-AMPK pathways during myricetin treatment of HCC cells. In addition, our study elucidated that autophagy induced by ER stress was protective against myricetin-induced cell death. Inhibition of autophagy enhanced the anti-HCC effect of myricetin. Our results showed that inhibiting autophagy by the chemical inhibitor chloroquine or knocking down ATG5 markedly enhanced myricetin-induced apoptosis (Figure 5). These results were further confirmed in a tumor orthotopic xenograft model in vivo (Figure 6). It has been reported that autophagy plays dual roles in HCC. Autophagy can serve as a tumor suppressor by inhibiting inflammation, p62 accumulation, the oxidative stress response, and maintaining genomic stability in normal cells [32]. However, another study demonstrated that autophagy promotes tumorigenesis and cancer cell survival in HCC by enhancing metabolism, inhibiting apoptosis and reactive oxygen species production, and increasing drug resistance [33]. Our results revealed that autophagy played a protective role in myricetin-induced apoptosis. Cotreatment with autophagy inhibitors improved the therapeutic effect of myricetin.

Clinically, for patients with early-stage HCC, liver transplantation, image-guided tumor ablation, resection, and transcatheter arterial chemoembolization (TACE) are widely accepted treatment options that improve the prognosis of HCC [6]. For patients with advanced-stage HCC, systemic therapies such as systemic chemotherapy with traditional cytotoxic drugs exhibit marginal activity and provide no survival benefit. From 2008 to 2017, sorafenib was the only agent proven to improve the outcome of advanced-stage HCC [9]. In 2017, the novel multikinase inhibitor regorafenib was shown to improve the survival of late-stage HCC patients as a second-line treatment [8]. Recently, lenvatinib has shown a noninferior survival benefit with sorafenib [10]. However, unsatisfactory survival improvement and the development of resistance against targeted therapy are the main obstacles to better prognosis. Thus, the exploration of novel potential therapeutics is in urgent demand. Natural compounds derived from plants are important sources of anticancer drugs [14]. For instance, paclitaxel, isolated from Taxus brevifolia, is now the first- or second-line treatment for many types of cancers [34]. Our data showed that as an active herbal agent, myricetin showed potent anti-HCC activity. However, before myricetin can be considered a potential anticancer agent, much work should be done to address several defects, such as its complex isolation procedures, poor water solubility, and low bioavailability [12]. These issues can be resolved by modifying the chemical structure, using drug-delivery carriers, combining with other drugs, etc. In fact, relevant work is already under way. M10, a derivative of myricetin that has good water solubility, can inhibit the development of ulcerative colitis and colorectal tumors [35]. In this study, we present a promising combination strategy: cotreatment with an autophagy inhibitor and myricetin to enhance the anti-HCC effect.

## 5. Conclusions

The current study demonstrated that myricetin suppressed the viability of HCC in vitro and in vivo. Our results indicated that apoptosis induced by myricetin in HCC could be triggered by ER stress and mediated by CHOP, which is a cell death activator during UPR. Myricetin also induced autophagy via ER stress in HCC cells through the IRE1*α*-JNK and Ca^2+^-AMPK pathways. Blockage of autophagy potentiated myricetin-induced apoptosis in HCC.

## Data Availability

The data that support the findings of this study are available from the corresponding author upon reasonable request.
